# Supplementary material for: From sequence to enzyme mechanism using multi-label machine learning
Source: BMC Bioinformatics. 2014 May 19;15:150. doi: 10.1186/1471-2105-15-150 (PMC4229970; doi:10.1186/1471-2105-15-150)
Supplement: Additional file 2 — Java code of ml2db. Additional file ml2db_code.tar.gz contains the Java source code to run the multi-label machine learning experiments and save the results to database. The code’s Javadoc is included. [file 1471-2105-15-150-S2.zip › additional file 2/ml2db/ml2db/doc/main/ml2db/mulan/learn/crosseval/CrossInputManager.html]

CrossInputManager


JavaScript is disabled on your browser.


- Overview
- Package
- Class
- Use
- Tree
- Deprecated
- Index
- Help

- Prev Class
- Next Class

- Frames
- No Frames

- All Classes

- Summary:
- Nested |
- Field |
- Constr |
- Method

- Detail:
- Field |
- Constr |
- Method


main.ml2db.mulan.learn.crosseval

## Class CrossInputManager

- java.lang.Object
- - main.ml2db.mulan.learn.crosseval.CrossInputManager

- Direct Known Subclasses:
  :   TrainTestInputManager

  ---

    

  ```
  public class CrossInputManager
  extends java.lang.Object
  ```

  An input manager for a single dataset (set of instances). Useful for cross
  evaluation.

  Version:
  :   15 Jul 2011

  Author:
  :   Luna De Ferrari ldf(at)st-andrews.ac.uk

- - ### Constructor Summary

    Constructors

    Constructor and Description

    `CrossInputManager(IdMultiLabelInstances trainSet,
    CrossExperimenter experiment)`

    Gets the instances as object.

    `CrossInputManager(int trainArffRecordId,
    DbManagers dbMan,
    CrossExperimenter crossExperimenter)`

    Gets the instances from database arff record.
  - ### Method Summary

    Methods

    | Modifier and Type | Method and Description |
    | --- | --- |
    | `void` | `checkTrainArffRecord()` |
    | `DbManagers` | `getDbManagers()` |
    | `CrossExperimenter` | `getExperiment()` |
    | `java.lang.String` | `getTrainArffPath()` |
    | `MulanArffRecord` | `getTrainArffRecord()` |
    | `IdMultiLabelInstances` | `getTrainDataSet()` |
    | `java.lang.String` | `getTrainXmlPath()` |
    | `void` | `setTrainDataSet(IdMultiLabelInstances trainDataSet)` |

    - ### Methods inherited from class java.lang.Object

      `equals, getClass, hashCode, notify, notifyAll, toString, wait, wait, wait`

- - ### Constructor Detail


    - #### CrossInputManager

      ```
      public CrossInputManager(IdMultiLabelInstances trainSet,
                       CrossExperimenter experiment)
      ```

      Gets the instances as object.

      Parameters:
      :   `trainSet` -


    - #### CrossInputManager

      ```
      public CrossInputManager(int trainArffRecordId,
                       DbManagers dbMan,
                       CrossExperimenter crossExperimenter)
      ```

      Gets the instances from database arff record.

      Parameters:
      :   `trainArffRecordId` -
      :   `arffRecordDbProps` -
  - ### Method Detail


    - #### checkTrainArffRecord

      ```
      public void checkTrainArffRecord()
      ```


    - #### getDbManagers

      ```
      public DbManagers getDbManagers()
      ```

      Returns:


    - #### getExperiment

      ```
      public CrossExperimenter getExperiment()
      ```


    - #### getTrainArffPath

      ```
      public java.lang.String getTrainArffPath()
      ```


    - #### getTrainArffRecord

      ```
      public MulanArffRecord getTrainArffRecord()
      ```


    - #### getTrainDataSet

      ```
      public IdMultiLabelInstances getTrainDataSet()
      ```


    - #### getTrainXmlPath

      ```
      public java.lang.String getTrainXmlPath()
      ```


    - #### setTrainDataSet

      ```
      public void setTrainDataSet(IdMultiLabelInstances trainDataSet)
      ```


- Overview
- Package
- Class
- Use
- Tree
- Deprecated
- Index
- Help

- Prev Class
- Next Class

- Frames
- No Frames

- All Classes

- Summary:
- Nested |
- Field |
- Constr |
- Method

- Detail:
- Field |
- Constr |
- Method
